# Physiological and Pathological Roles of CaMKII-PP1 Signaling in the Brain

**DOI:** 10.3390/ijms19010020

**Published:** 2017-12-22

**Authors:** Norifumi Shioda, Kohji Fukunaga

**Affiliations:** 1 Department of Biofunctional Analysis Laboratory of Molecular Biology, Gifu Pharmaceutical University, 1-25-4 daigaku-nishi, Gifu 501-1196, Japan; 2 Department of Pharmacology, Graduate School of Pharmaceutical Sciences, Tohoku University, 6-3 Aramaki-Aoba, Aoba-ku, Sendai, Miyagi 980-8578, Japan

**Keywords:** Ca^2+^/calmodulin-dependent protein kinase II, protein phosphatase-1, synaptic plasticity, nuclear translocation

## Abstract

Ca^2+^/calmodulin (CaM)-dependent protein kinase II (CaMKII), a multifunctional serine (Ser)/threonine (Thr) protein kinase, regulates diverse activities related to Ca^2+^-mediated neuronal plasticity in the brain, including synaptic activity and gene expression. Among its regulators, protein phosphatase-1 (PP1), a Ser/Thr phosphatase, appears to be critical in controlling CaMKII-dependent neuronal signaling. In postsynaptic densities (PSDs), CaMKII is required for hippocampal long-term potentiation (LTP), a cellular process correlated with learning and memory. In response to Ca^2+^ elevation during hippocampal LTP induction, CaMKIIα, an isoform that translocates from the cytosol to PSDs, is activated through autophosphorylation at Thr286, generating autonomous kinase activity and a prolonged Ca^2+^/CaM-bound state. Moreover, PP1 inhibition enhances Thr286 autophosphorylation of CaMKIIα during LTP induction. By contrast, CaMKII nuclear import is regulated by Ser332 phosphorylation state. CaMKIIδ3, a nuclear isoform, is dephosphorylated at Ser332 by PP1, promoting its nuclear translocation, where it regulates transcription. In this review, we summarize physio-pathological roles of CaMKII/PP1 signaling in neurons. CaMKII and PP1 crosstalk and regulation of gene expression is important for neuronal plasticity as well as survival and/or differentiation.

## 1. Introduction

Protein phosphorylation, one of the most important post-translational modifications, drives rapid, reversible and extracellular signal-dependent cell signaling. In the brain, Ca^2+^/calmodulin (CaM)-dependent protein kinase II (CaMKII), a multifunctional serine (Ser) and threonine (Thr) kinase [1], regulates diverse Ca^2+^-mediated neuronal activities, including neurotransmitter release, gene expression, and synaptic plasticity [2,3]. CaMKII is a dodecameric holoenzyme assembled from α, β, γ, and δ isoforms. In eukaryotes, these four CaMKII isoforms are encoded by distinct genes, and their corresponding mRNAs are alternatively spliced to give rise to subtypes exhibiting variable domains [4,5].

CaMKII has attracted substantial attention due to its function in synaptic plasticity, an activity that occurs at postsynaptic densities (PSDs) [6,7,8]. In response to Ca^2+^ elevation by extracellular stimuli, Ca^2+^-CaM binding to CaMKII displaces autoinhibitory domains to allow ATP and exogenous substrates access to the active site. Immediately after activation, Thr286 in the autoinhibitory domain of the α isoform (corresponding to Thr287 of β, γ, and δ isoforms) is autophosphorylated by the neighboring kinase domain. This event increases Ca^2+^-CaM binding affinity and blocks interaction of autoinhibitory and catalytic domains, thereby generating autonomous kinase activity and prolonging the Ca^2+^/CaM-bound state. CaMKIIα autonomy is critical for induction and maintenance of hippocampal long-term potentiation (LTP), both of which underlie learning and memory [9,10].

The 12 subunits of the CaMKII holoenzyme assemble into two coplanar rings, each containing six subunits [11]. These ring structures suggest a potential mechanism for establishment of an autonomous kinase state, as it is proposed that autophosphorylation occurs by an inter-subunit process [12]. Indeed, following a robust and long Ca^2+^ stimulus, two adjacent CaMKII monomers are simultaneously bound by Ca^2+^/CaM. In these conditions, one subunit serves as a substrate for the other, resulting in Thr286/Thr287 phosphorylation. Once the first subunit is phosphorylated, subsequent phosphorylation within the holoenzyme is more likely to occur, as lower Ca^2+^ levels are required for the second phosphorylation. Thus, in this scenario, CaMKII remains active, even when Ca^2+^ levels return to basal levels, until it is dephosphorylated. If the number of phosphorylated subunits exceeds a threshold and the phosphorylation rate is greater than the dephosphorylation rate, then CaMKII activity is sustained [13].

In contrast to postsynaptic CaMKII function, the physiological relevance of nuclear activity of CaMKII isoforms in the central nervous system (CNS) remains unclear. Alternative splicing of CaMKII generates a multitude of isoforms for each CaMKII subunit [5]. Among these alternatively splicing isoforms, CaMKIIαB [14], CaMKIIγA [15], and CaMKIIδ3 (also called CaMKIIδB) [16] display consensus (KKRK) sequences in respective variable domains that resemble a nuclear localization signal (NLS) and are homologous to the simian virus 40 (SV40) large T antigen NLS [17]. In rat brain neurons, CaMKIIαB and CaMKIIδ3 are expressed in the nucleus [14,18], and their activity is reportedly regulated by the NLS motif, which, when phosphorylated, prevents nuclear localization. CaMKIIδ3 Ser332, which is immediately C-terminal to the NLS (^328^KKRKS^332^), is reportedly phosphorylated by the CaMK family members CaMKI or CaMKIV, blocking association of CaMKII with the NLS receptor m-pendulin and thereby preventing nuclear localization [19].

The Ser/Thr phosphatase Protein Phosphatase-1 (PP1), a key regulator of CaMKII signaling, forms a heterodimer comprised of a catalytic (PP1c) and a regulatory subunit. PP1c can form a complex with over 50 regulatory or scaffolding proteins that dictate substrate specificity and subcellular localization [20]. In mammalian cells, PP1c itself occurs as different isoforms (α, β, δ, γ1 and γ2) [21,22,23,24,25], and three (PP1α, PP1β, and PP1γ1) are highly expressed in the brain [26]. All isoforms show nearly 90% amino acid homology and are most divergent at the N- and C-termini. Importantly, although CaMKIIα Thr286 can be dephosphorylated by PP1, PP1 appears to play a more prominent role in CaMKII dephosphorylation at PSDs [27]. Moreover, CaMKIIδ3 is dephosphorylated at Ser332 by PP1, promoting its nuclear translocation [28].

In this review, we focus on the role of CaMKII/PP1 signaling in both neuronal plasticity at PSDs and gene expression in the nuclei. We also discuss how imbalanced CaMKII/PP1 activity may underlie neuronal pathologies, such as mental disorders and neurodegeneration.

## 2. Physiological Function of CaMKII/PP1 Signaling at PSDs

PSDs are localized in the tips of dendritic spine heads and contain multiple classes of proteins that function in neuronal signaling in response to presynaptic neurotransmitter release, such as glutamate [29]. CaMKII is one of the most abundant proteins found in forebrain PSDs [30]. CaMKII regulates synaptic strength, in part by phosphorylating glutamate receptors [31]. As noted, CaMKIIα Thr286 autophosphorylation promotes autonomous kinase activity, which when sustained is essential for learning and memory [9,10]. Thus, it is critical to understand how CaMKII remains highly phosphorylated and resists endogenous phosphatase activity.

One factor governing this persistent “on-state” is PP1 localization to dendritic spines and PSDs [32,33]. PP1 inhibition enhances CaMKIIα Thr286 autophosphorylation during LTP induction [34]. However, autophosphorylated CaMKIIα Thr286 cannot be dephosphorylated by PP1 in purified PSDs from rats [35]. These authors also showed that the Thr286 site is not buried within the CaMKIIα protein, as it can be dephosphorylated in purified PSDs by exogenous soluble PP1c or λ phosphatase. These results indicate that the inability of PP1 to dephosphorylate this site in vivo is due to the positioning of PP1 in PSDs and the inhibitory activity of scaffolding proteins that modulate PP1 activity [36,37]. For example, spinophilin and its homolog neurabin are F-actin binding proteins that target PP1 to PSDs, and spinophilin alters PP1 catalytic activity by steric inhibition of substrate binding sites [38]. Indeed, numerous protein-protein interactions hold PP1 in such a position that PP1 simply cannot reach CaMKIIα Thr286.

Once activated, CaMKII remains in an active conformation throughout the LTP maintenance phase, an observation that forms the basis of the hypothesis that CaMKII is critical for memory formation [9,10]. However, these conclusions are based on work carried out using hippocampal homogenates, and these studies do not provide specific information relevant to the pool of active CaMKII at synapses. Some studies using Camui, a fluorescence resonance energy transfer (FRET)-based CaMKII sensor [39], show that CaMKII activity lasts only ~1 min after stimulation during LTP induction, based on two-photon laser-mediated photolysis of caged glutamate at hippocampal CA1 spines [40,41]. CaMKII activity, as measured by the magnitude of the Camui-FRET change, was not affected by treatment with Calyculin A, a PP1/PP2A phosphatase inhibitor [41]. Thus, optical monitoring of CaMKII activity has the advantage of greater spatiotemporal resolution over previous immunoblotting studies. However, they still have technical limitations relating to their ability to detect small amounts of activated CaMKII within dendritic spines. Thus, the relationship between LTP and CaMKII/PP1 signaling needs further investigation.

## 3. Pathological CaMKII/PP1 Signaling in PSDs

In animal models of Parkinson’s disease, striatal dopamine depletion increases CaMKIIα autophosphorylation at Thr286 in parallel with decreased PP1γ1 activity and increased PP1γ1 binding to spinophilin [42,43,44]. Moreover, we showed increased CaMKIIα Thr286 autophosphorylation and decreased levels of spinophilin and PP1 in the prefrontal cortex of a mouse model of α-thalassemia X-linked mental retardation (ATR-X) syndrome [45]. This pathological imbalance of CaMKII/PP1 signaling in the ATR-X model correlated with altered dendritic spine morphology, suggesting that CaMKII/PP1 signaling regulates this process [45]. Likewise, decreased PP1 activity in the brain of Angelman syndrome model mice correlated with increased phosphorylation of hippocampal CaMKIIα at Thr286 in PSDs, as well as with changes in synaptic plasticity, learning, and memory [46]. This evidence suggests overall that increased CaMKII activity is mediated by reduced PP1 activity, particularly in PSDs, thereby perturbing synaptic plasticity and learning and memory.

## 4. Physiological CaMKII/PP1 Signaling in Nuclei

Transduction of signals from synapses to the nucleus is primarily mediated by Ca^2+^ signaling, and nuclear Ca^2+^ transients are some of the most potent regulators of neuronal gene expression [47]. Nuclear CaMKII transcriptionally regulates the gene encoding neurotrophin brain-derived neurotrophic factor (BDNF) [48,49] through phosphorylation of diverse nuclear proteins, including cAMP response element-binding protein (CREB) [50,51], methyl CpG binding protein 2 (MeCP2) [52], activating transcription factor [53,54], CCAAT/enhancer-binding protein [55,56], and serum response factor [57].

Specifically, CaMKII phosphorylates CREB at Ser133 and Ser142 in vitro [51]. Moreover, Ca^2+^-induced CaMKII activation in primary cultured neurons stimulates CREB phosphorylation at Sers 133, 142, and 143 [58]. CREB phosphorylation at Ser142 and Ser143 contributes to its activation, and alanine mutations at Ser142 and Ser143 block Ca^2+^-induced CREB-dependent transcription [58]. However, transgenic mice harboring a single CREB Ser142-to-alanine mutation show alterations in the circadian clock located in the suprachiasmatic nucleus, which down-regulate c-Fos, a transcriptional target of CREB [59]. The transcription factor MeCP2 binds to methylated cytosine residues of CpG dinucleotides in DNA [60]. Neuronal activity and subsequent Ca^2+^ influx trigger CaMKII-dependent MeCP2 phosphorylation at Ser421 [52]. Knock-in mice that lack MeCP2 Ser421 or Ser421 and Ser424, a second site of synaptic activity-induced phosphorylation, show perturbed synaptogenesis, synaptic plasticity, and spatial memory [61,62], underscoring the importance of these phosphorylation sites in vivo.

Until recently, mechanisms underlying substrate phosphorylation by nuclear CaMKII remained unclear. Thus, we investigated nuclear-cytoplasmic shuttling of the nuclear isoform CaMKIIδ3. Previously, others had reported that CaMKIIδ3 Ser332, which is C-terminal to the NLS (^328^KKRKS^332^), is phosphorylated by CaMKI or CaMKIV, prohibiting nuclear localization [19]. To investigate a potential function of CaMKII phosphorylation, we generated a specific antibody against phosphorylated Ser332 of CaMKII. In an in vitro phosphorylation assay of purified rat brain CaMKII, CaMKIIδ3 was dephosphorylated by PP1 at both Ser332 and Thr287 [28]. We also showed that PP1α and PP1γ1 predominantly regulate CaMKIIδ3 nuclear translocation in Neuro-2a cells. However, nuclear CaMKIIδ3 activity in Neuro-2a cells was enhanced by PP1γ1 overexpression. Consistent with these results, in experiments using primary cultured mesencephalic dopamine neurons, CaMKIIδ3 was dephosphorylated only at Ser332, not at Thr287, by activated PP1 [28]. This discrepancy may be explained by the binding of various proteins to the CaMKII/PP1 complex, in a manner similar to spinophilin in PSDs. We conclude that the in vitro experimental conditions used in our study resemble the cytosolic microenvironment, in which PP1 directly dephosphorylates cytosolic CaMKIIδ3. We have not yet defined proteins binding to and regulating the CaMKIIδ3/PP1 complex in vivo, an analysis that awaits future studies.

Others have reported nuclear activity of CaMKIIαB and CaMKIIγA in neurons [63,64]. For example, in rat retinal ganglion cells CaMKIIαB expression and nuclear translocation increase via an unknown mechanism following glutamate-induced cell death [63]. Ma et al. also reported that CaMKIIγA functions as a transporter of Ca^2+^/CaM to the nucleus following depolarization of cultured superior cervical ganglion neurons and that the Ca^2+^/CaM-CaMKIIγ complex is dephosphorylated at Ser334 by calcineurin, allowing it to shuttle to the nucleus. Nuclear delivery of Ca^2+^/CaM activates nuclear CaM kinases, including CaMKIV and CaMKK, driving CREB phosphorylation and transcription of its target genes [64]. Therefore, phosphatases other than PP1, such as calcineurin and/or PP2A, may dephosphorylate Ser332 of CaMKIIδ3 in other types of neurons.

## 5. Pathological CaMKII/PP1 Signaling in Nuclei

CaMKII-PP1 signaling transcriptionally regulates BDNF, a factor vital for neuronal survival, growth, and maintenance, in brain circuits functioning in emotion and cognition [65]. MeCP2 mutations cause most cases of Rett syndrome, an X-linked dominant neurodevelopmental disorder and a leading cause of mental retardation and autistic behavior in females [66]. Phenotypes, such as normal early development followed by progressive motor and cognitive dysfunction, seen in mice that either lack or overexpress MeCP2 recapitulate many characteristic features of Rett syndrome [67,68,69]. In addition, like syndrome patients, MeCP2 mutant mice show abnormalities in brain morphology and cyto-architecture, in particular a decrease in dendritic arborization and spine loss [52,70]. Importantly, MeCP2 Ser421 phosphorylation by CaMKII is required for activity-dependent regulation of BDNF gene expression [52], suggesting that transcriptional deregulation of this gene potentially due to CaMKII dysregulation plays a central role in Rett syndrome.

We also previously revealed that nuclear CaMKII/PP1 signaling is important for neuronal survival and differentiation [28]. We reported that the nuclear isoform CaMKIIδ3 is highly expressed in dopaminergic rat substantia nigra neurons [71] and that stimulation of the dopamine D2 receptor (D2R) activates CaMKIIδ3, inducing BDNF gene expression in NG108-15 cells [72]. We also found that CaMKIIδ3 Ser332 is directly dephosphorylated by PP1, promoting CaMKIIδ3 nuclear translocation, and that aripiprazole (APZ), a dopamine D2R partial agonist, promotes CaMKIIδ3 nuclear translocation and enhances BDNF expression [28]. APZ treatment also enhanced sprouting and survival of cultured dopaminergic neurons through the CaMKIIδ3/PP1 pathway [28]. Consistent with our results, APZ treatment for eight weeks was reported to significantly increase plasma BDNF levels in first-episode untreated schizophrenia patients [73]. BDNF protein expression decreases in the dopamine-deficient substantia nigra of Parkinson disease patients [74,75]. BDNF also reportedly promotes survival of cultured mesencephalic dopaminergic neurons [76] and, in vivo, protects dopaminergic neurons from damage by the neurotoxins 1-methyl-1,2,3,6-tetrahydropiridine and 6-hydroxydopamine [77]. This evidence and our data suggest a critical role for BDNF in supporting survival and/or differentiation of midbrain dopaminergic neurons functioning nuclear CaMKII/PP1 pathway with the APZ treatment.

## 6. Conclusions

**Figure 1:**
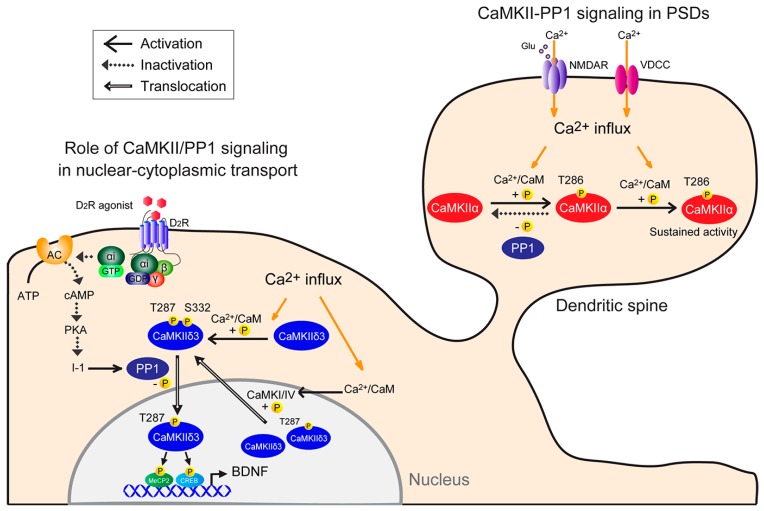
Model of neuronal CaMKII-PP1 signaling. (1) CaMKII-PP1 signaling in PSDs: CaMKII is simultaneously bound by Ca^2+^/CaM following a Ca^2+^ stimulus. In this condition, one subunit acts as a substrate for the other, resulting in Thr286 phosphorylation. Once that subunit is phosphorylated, subsequent phosphorylation within the holoenzyme is more likely to occur, as Ca^2+^ levels required for the second phosphorylation are lower than those required for the initial phosphorylation (sustained activity). Thus, CaMKII remains active, even when basal Ca^2+^ levels are re-established, until it is dephosphorylated by PP1. CaMKII activity is sustained if the number of phosphorylated subunits exceeds a threshold and the phosphorylation rate exceeds the dephosphorylation rate. (2) Role of CaMKII/PP1 signaling in nuclear-cytoplasmic transport: Under basal conditions, CaMKIIδ3 is autonomously active in part due to spontaneous neuronal activity. Cytoplasmic CaMKIIδ3 is autophosphorylated, and D2R-mediated PP1 activation mediates CaMKIIδ3 dephosphorylation at Ser332. For example, stimulation with a dopamine D2R agonist increases PP1 activity by inactivating the cAMP/PKA/inhibitor 1 (I-1) pathway, and in turn PP1 dephosphorylates CaMKIIδ3 at Ser332 in the cytoplasm, enabling its nuclear translocation. Thereafter, nuclear CaMKII3 phosphorylates transcription factors, including MeCP2 and CREB, increasing BDNF expression. Depolarization causes Ca^2+^ entry into neurons through NMDA receptors or voltage-dependent calcium channels and promotes CaMKIIδ3 autophosphorylation at Thr287 and Ser332 in the cytosol. Conversely, nuclear CaMKI or CaMKIV activity may promote CaMKIIδ3 nuclear export via Ser332 phosphorylation.

CaMKII/PP1 signaling plays a crucial role in many different aspects of synaptic plasticity in PSDs and in activity-regulated transcription in nuclei. CaMKII alternative splicing generates numerous subtypes of each CaMKII isoform. Figure 1 summarizes how each function, in relationship to others, mediates Ca^2+^ signaling to PSDs or nuclei. However, the composition of the dodecameric CaMKII holoenzyme affects CaMKII localization [78,79]. The ability of CaMKII to translocate to the nucleus is thus governed by the presence of nuclear versus cytoplasmic isoforms that make up holoenzyme [17]. Nuclear CaMKII isoforms containing an NLS (CaMKIIαB, CaMKIIδ3, and CaMKIIγA) may co-assemble with cytoplasmic subunits, including postsynaptic density-associated CaMKIIα [80] and/or F-actin-associated CaMKIIβ [81] to facilitate synaptic activation or nuclear translocation. Further study is required to reveal the relationship between oligomerization of heterogenous CaMKII isoforms and PP1 in neurons.

## Abbreviations

AC	adenylate cyclase
ATR-X	α-thalassemia X-linked mental retardation
BDNF	brain-derived neurotrophic factor
CaM	calmodulin
CaMKII	Ca^2+^/calmodulin-dependent protein kinase II
cAMP	cyclic adenosine monophosphate
CNS	central nervous system
CREB	cAMP response element-binding protein
D2R	dopamine D2 receptor
LTP	long-term potentiation
MeCP2	methyl CpG binding protein 2
NLS	nuclear localization signal
NMDA	*N*-methyl-d-aspartate
PKA	protein kinase A
PP1	Protein Phosphatase-1
PSDs	postsynaptic densities
Ser	serine
SV40	simian virus 40
Thr	threonine
VDCC	voltage-dependent calcium channel
